# Mesothelioma and Social Security Benefits in Brazil: 2019 to 2021

**DOI:** 10.47626/1679-4435-2025-1448

**Published:** 2025-11-17

**Authors:** Luiz Eduardo Fonseca e Gomes, Norma Suely Souto Souza

**Affiliations:** 1 Escola Bahiana de Medicina e Saúde Pública, Salvador, BA, Brazil.; 2 Ministério da Previdência Social, Divisão Regional da Perícia Médica Federal 25, Salvador, BA, Brazil.

**Keywords:** asbestos, mesothelioma, social security, amianto, mesotelioma, previdência social

## Abstract

**Introduction:**

Asbestos is carcinogenic to humans, according to the International Agency for Research on Cancer of the World Health Organization, and can cause several diseases, including mesothelioma, a malignant tumor of the pleura and peritoneum, mainly.

**Objectives:**

Analysis of disability pension benefits with mesothelioma diagnosis in Brazil from 2019 to 2021.

**Methods:**

Descriptive study using records of benefits granted by the Social Security National Institute (Instituto Nacional do Seguro Social) in Brazil to insured workers under the Regime Geral da Previdência Social with a diagnosis of mesothelioma or pleural cancer. Later, cases were identified as mesothelioma after reviewing medical-pericial reports. Two Instituto Nacional do Seguro Social data sources were used: Unified Benefits Information System, providing sociodemographic data of the insured and benefit information, and Disability Benefits Administration System (SABI), where the medical-pericial report is prepared, providing information on the insured’s occupation and the type of mesothelioma.

**Results:**

During the study period, 109 benefits were granted by Instituto Nacional do Seguro Social with a mesothelioma diagnosis, and six with a pleural cancer diagnosis, which upon review of the medical-pericial report were identified as mesothelioma. Of the total 115 benefits granted, only one was characterized as occupational.

**Conclusions:**

Only one benefit was characterized as occupational disease, contrary to the literature evidence of mesothelioma being associated with occupational or environmental asbestos exposure in 80% of cases.

## Abstract (pt)

**Introdução:**

O amianto é cancerígeno para humanos, segundo a Agência Internacional de Pesquisa em Câncer da Organização Mundial da Saúde, e pode provocar diversas doenças, entre elas, o mesotelioma, um tumor maligno de pleura e peritônio, principalmente.

**Objetivos:**

Analisar os benefícios previdenciários por incapacidade com diagnóstico de mesotelioma no Brasil, no período compreendido entre 2019 e 2021.

**Métodos:**

Estudo descritivo que usou registros de benefícios concedidos pelo Instituto Nacional do Seguro Social no Brasil a trabalhadores segurados do Regime Geral da Previdência Social com diagnóstico de mesotelioma ou de câncer de pleura, que posteriormente foram identificados como casos de mesotelioma após revisão dos laudos médico-periciais. Foram usadas duas fontes de dados do Instituto Nacional do Seguro Social: o Sistema Único de Informações de Benefícios, que fornece dados sociodemográficos do segurado e informações sobre o benefício; e o Sistema de Administração de Benefícios por Incapacidade, no qual é elaborado o laudo médico-pericial, de onde foram coletadas informações sobre a ocupação do segurado, bem como o tipo de mesotelioma.

**Resultados:**

No período de estudo, foram concedidos 109 benefícios pelo Instituto Nacional do Seguro Social com diagnóstico de mesotelioma e seis com diagnóstico de câncer de pleura, cuja revisão do laudo médico-pericial mostrou tratar-se de mesotelioma. Do total de 115 benefícios concedidos, apenas um foi caracterizado como ocupacional.

**Conclusões:**

Apenas um benefício foi caracterizado como doença ocupacional, o que vai de encontro às evidências da literatura de associação do mesotelioma à exposição ocupacional ou ambiental ao amianto em 80% dos casos.

## INTRODUCTION

Asbestos is a low-cost mineral widely used in construction (eg, water tanks, vinyl flooring, ceilings, and piping) and in other sectors as an insulating material for gaskets, packings, sealing components, clutch discs, specialty fabrics, paints, and in the defense, aerospace, oil, paper, shipbuilding, and foundry industries. It is valued for its flame resistance, strong insulating capacity, durability, flexibility, indestructibility, resistance to acids, alkalis, and bacteria, and its ease of weaving, among other properties.^1^

Despite these industrial qualities, asbestos is carcinogenic to humans, according to the International Agency for Research on Cancer of the World Health Organization. It can cause several diseases, including asbestosis and cancers of the lung, larynx, and ovary as well as mesothelioma — a malignant tumor that arises primarily in the pleura and, less often, in the peritoneum.^1,2^

Mesothelioma is a rare cancer associated with occupational or environmental exposure to asbestos in about 80% of cases.^3^ The pleura is the most common primary site (82%), followed by the peritoneum (9%).^4^ The disease predominantly affects men, and risk increases with age, being more prevalent among individuals older than 65 years.^5^ The average latency between the exposure to asbestos and the diagnosis of mesothelioma is roughly 30 years.^6^ Median survival is 9 months for pleural mesothelioma and 18 months for non-pleural mesothelioma.^7^

Brazil — historically one of the world’s major producers and exporters of asbestos6 — recorded 3,057 deaths from asbestos-related diseases between 1996 and 2017, of which 2,405 (76.4%) were mesotheliomas.^8^ In November 2017, a nationwide ban on the use of asbestos was imposed by Brazil’s Federal Supreme Court (STF). However, because of the long latency of mesothelioma and the delayed timeline for phasing out asbestos in Brazil, incidence peaks are still expected in the coming decades. In addition, maintenance workers — especially in the informal sector — are likely to remain at risk of exposure.

Brazilian social-security legislation defines work- related accidents in Articles 19-23 of Law No. 8,213/1991 and classifies “occupational disease” as that produced or triggered by work inherent to a specific activity and listed by the Ministry of Labor and Social Security. Work-related disease is defined as that acquired or triggered by special conditions under which the work is performed and that is directly related to it; such conditions are also listed by the Ministry.^9^

Insured workers with work-related cancer are entitled to recognition of harm and specific compensation (eg, retirement, pensions, and allowances) and other compensatory mechanisms when a causal link between the cancer and the work is established.^10^ The technical work-related nexus is determined by associating the health outcome with etiologic agents or risk factors present in the employer’s economic activities.

Decree No. 3,048/1999 of the Ministry of Social Security lists, in Annex II, diseases originating in work processes, organized into Lists A and B. List A links occupational agents or risk factors to the etiology of occupational/work-related diseases, and List B enumerates diseases together with their occupational etiologic agents or risk factors. Asbestos appears on these lists; among the related diseases are mesotheliomas of pleura (C45.0), peritoneum (C45.1), and pericardium (C45.2).^11^

In practice, establishing a causal link to work is often difficult because of the disease’s long latency and other factors, including poor-quality and underreported data; a scarcity of studies; limited public and professional awareness of the issue; lack of worker organization to claim rights; and low rates of litigation in mesothelioma cases.^12^

The current study aims to analyze social-security disability benefits granted to insured workers with a diagnosis of mesothelioma in Brazil between 2019 and 2021.

## METHODS

We conducted a descriptive study using records of disability benefits granted by Brazil’s Instituto Nacional do Seguro Social (INSS, in Portuguese) to insured workers covered by the General Social Security Regime (RGPS, in Portuguese) with a diagnosis of mesothelioma from January 1, 2019, to December 31, 2021.

The study population comprised all workers insured by Brazil’s social security system. Benefits were selected when the diagnosis field contained mesothelioma (International Classification of Diseases, 10th revision [ICD-10]: C45.0, C45.1, C45.2, C45.7, C45.9) or pleural cancer (ICD- 10 C38.4) with a benefit start date within the study period.

A total of 2 data sources from the INSS were used: 1) the Unified Benefits Information System (SUIBE, in Portuguese), a registry that provides formatted tables and reports with sociodemographic characteristics, diagnosis, and benefit information; and 2) the Disability Benefits Administration System (SABI, in Portuguese), which contains the medical- expert report, from which we obtained the insured worker’s occupation, diagnosis, and mesothelioma type (anatomic site).

Although SABI includes a specific field for occupation, for this study we used the occupation described in the “clinical history” field because, according to federal medical examiners, it is completed more systematically. The mesothelioma diagnosis was also verified in the “clinical history” to confirm the accuracy of the ICD code entered in the “diagnosis” field, given the possibility of miscoding. When the “diagnosis” field listed pleural cancer, the “clinical history” was likewise reviewed to determine whether mesothelioma was indicated. Diagnoses by federal medical examiners are based on documentation submitted by the insured during the exam (attestation/report from the treating physician and complementary tests).^13^

Table and report generation in SUIBE and data extraction from SABI medical-expert reports were performed by one of the authors, a physician with the federal medical examiner service. Access to these systems and use of data for research were authorized by the INSS Executive Management Office in Salvador.

We analyzed the following variables for insured workers: sex; age (in years, described as a mean and by age-group categories); type of INSS affiliation (employee, domestic employee, casual port worker, special insured worker, and voluntary contributor); and occupation. For benefits, we assessed the type: temporary disability benefit — social-security; temporary disability benefit — work-related; permanent disability benefit — social-security; and permanent disability benefit — work-related.

Frequencies, percentages, and/or means were calculated to characterize the population and the benefits granted. Data were tabulated and analyzed using Microsoft Excel.

The research protocol was submitted to the Research Ethics Committee at Escola Bahiana de Medicina e Saúde Pública via Plataforma Brasil and approved under CAAE 66829022.2.0000.5544, opinion 5,912,144, in February 2023.

## RESULTS

Between 2019 and 2021, the INSS granted 123 benefits with a diagnosis of mesothelioma; 14 were excluded after the “clinical history” in the SABI medical-expert report failed to confirm the diagnosis. In addition, 62 benefits were granted with a diagnosis of pleural cancer, 6 of which were described as mesothelioma in the clinical history. In total, 115 benefits were granted for mesothelioma during the period — 109 coded as ICD-10 C45 (mesothelioma) and 6 as ICD-10 C38.4 (pleural cancer).

Regarding the sociodemographic and social- security characteristics of insured workers who received these benefits, females predominated (56.5%), and the mean age was 53 years. The most common INSS affiliation was employee (44.3%), followed by self-employed (30.4%). The most frequent occupations were drivers (7.0%); commercial manager and teacher (6.1% each); salesperson, business owner, and homemaker (5.2% each). Occupation was not reported for 13.9% of benefits (Table 1).

**Table 1:** Distribution of social security benefits granted for mesothelioma by sociodemographic and social-security characteristics of insured workers — Brazil, 2019-2021 (N = 115)

Variable	n	%	Mean
Sex		
Male	50	43.5
Female	65	56.5
Age		53 ± 10
20-29 years	1	0.9
30-39 years	20	174
40-49 years	27	23.5
50-59 years	44	38.3
60-69 years	23	20.0
Type of INSS affiliation OK		
Unemployed	21	18.3
Employed	51	44.3
Domestic worker	2	1.7
Voluntary contributor	2	1.7
Special insured worker	4	3.5
Self-employed	35	30.4
Occupation		
Driver	8	7.0
Teacher	7	6.1
Commercial manager	7	6.1
Salesperson	6	5.2
Business owner	6	5.2
Homemaker	6	5.2
General services assistant	6	5.2
Domestic worker/house cleaner	5	4.3
Administrative manager	4	3.5
Maintenance supervisor	4	3.5
Checker	3	2.6
Cook/kitchen helper	3	2.6
Administrative assistant	3	2.6
Other	31	26.9
Not reported	16	13.9

Source: Unified Benefits Information System (SUIBE), Instituto Nacional do Seguro Social (INSS) and Disability Benefits Administration System, INSS.

**Figure 1:**
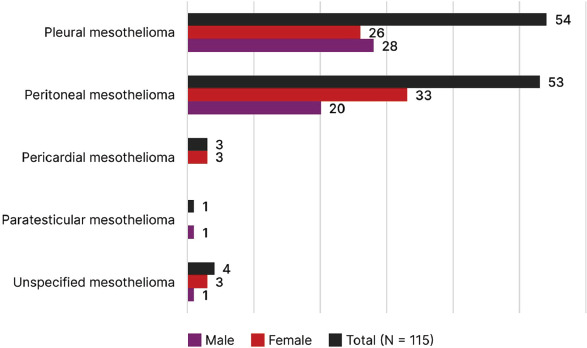
Distribution of social security benefits granted for mesothelioma by body site and sex — Brazil, 2019-2021 (N = 115). Source: Disability Benefits Administration System, Instituto Nacional do Seguro Social.

With regard to the affected body site, there was virtually no difference between the pleura (47.0%) and the peritoneum (46.1%). Among insured workers with pleural mesothelioma, males predominated (51.9%), whereas among those with peritoneal mesothelioma, females predominated (62.3%) (Figure 1).

Nearly all benefits (114) were non-occupational, with temporary disability benefits predominating (83.5%), followed by permanent disability retirement (15.7%). Only 1 benefit was classified as occupational (work-related).

## DISCUSSION

During the study period, the INSS granted 109 benefits with a diagnosis of mesothelioma (C45.X) and 6 with a diagnosis of pleural cancer (C38.4) that, upon review, corresponded to mesothelioma. In ICD-10, code C45 was created to specify malignant mesothelioma.^14^ Despite this new coding, previous studies showed that deaths coded as pleural cancer (C38.4) could be used to estimate mesothelioma cases (C45.0).14 Accordingly, we included C38.4 when identifying benefits granted in the period. However, after checking the clinical history in the medical- expert reports, only 9.7% of diagnoses of pleural cancer (C38.4) were actually pleural mesothelioma (C45.0).

Although mesothelioma linked to asbestos exposure is primarily associated with male- dominated work activities, most benefits in this study (56.5%) were granted to women — unlike findings elsewhere. In Italy, 28.5% of incident mesothelioma cases occurred in women,^15^ and in Brazil, mortality analyses reported 41.7% of mesothelioma deaths among women.^8^ Relevant occupational exposures for women include jobs with direct asbestos use as well as indirect exposures from asbestos present in workplace structures and machinery.15 Additional exposure pathways include domestic and environmental exposure and other unidentified routes.^15^ Environmental exposure has been associated with residences near industries that use asbestos,^16^ while domestic exposure may arise from handling the contaminated clothing of partners employed in such industries or from paid work washing miners’ uniforms.^15,16^

One possible explanation for the predominance of benefits granted to women in our study is the longer survival observed among women with peritoneal mesothelioma. Women with this tumor type tend to live longer than men after diagnosis (13 vs 6 months),^17^ which could allow multiple temporary benefits to be granted to the same individual. Diagnostic error in peritoneal mesothelioma is another possibility. Typical signs and symptoms include abdominal pain, increased abdominal girth, ascites, asthenia, and weight loss. Computed tomography may show ascites, peritoneal thickening, omental caking, solid tumors invading intra-abdominal organs, and diaphragmatic involvement.^18,19^ None of these clinical or imaging findings is specific for this rare disease. Diagnosis is complex and requires review by an experienced pathologist and use of broad immunohistochemical panels, since no single stain is pathognomonic for peritoneal mesothelioma.17 Consequently, the disease may be mistaken for its main differential diagnoses, including peritoneal carcinomatosis, primary peritoneal serous carcinoma, ovarian carcinoma, lymphomatosis, and peritoneal tuberculosis.^20^

With respect to age at presentation, mesothelioma is indeed rare before 30 years of age because of its long latency, which is consistent with the age distribution observed in this study. However, the proportion of benefits granted to individuals aged 30-39 years — 17.4% of all benefits — warrants attention. Given how young this group is for a disease with such a prolonged latency, early-life asbestos exposure is a plausible hypothesis. Additionally, younger insured individuals may carry germline mutations in cancer-predisposition genes. Such mutations are more frequent in younger patients and in those with little or no asbestos exposure.21 Up to 12% of patients diagnosed with mesothelioma harbor these mutations, most commonly in *BAP1*.^21^

Regarding occupation, many insured individuals were not engaged in industrial or mining activities. Because mesothelioma is considered the principal marker of asbestos exposure in a society^22^ — generally of occupational origin — one hypothesis is that federal medical examiners documented only the insured’s current occupation, without retrieving prior occupational histories involving asbestos exposure, a data point with which many health professionals are unfamiliar.^22^ Another possibility is para-occupational exposure — ie, working in places where asbestos is present without directly handling it.^22^

In the literature, the pleura is widely recognized as the most commonly affected site, accounting for most cases, followed by the peritoneum.^4^ Our findings differ from this pattern: there was virtually no difference in the number of benefits granted for pleural versus peritoneal mesothelioma (54 vs 53). The explanations likely mirror those proposed above for the predominance of benefits among women, since most insured individuals with peritoneal mesothelioma in our sample were female: women with peritoneal mesothelioma have longer survival, enabling multiple social-security benefits to be granted to the same individual over time; diagnostic error in peritoneal mesothelioma may also play a role.

Self-employed workers, domestic workers, and voluntary contributors do not have their health conditions assessed for occupational etiology by the medical examiner service due to legal provisions or the INSS operating system. Consequently, 42 of the 115 mesothelioma-related benefits were not evaluated for potential occupational causation. Among the 73 benefits assessed for an occupational nexus, only 1 was classified as occupational — likely reflecting the difficulty of establishing causality given the disease’s long latency. Latency complicates diagnosis, as workers may have recall bias and fail to remember prior occupational exposures. In 1 study of medical records for patients diagnosed with pleural mesothelioma between 2009 and 2020, only 41% were aware of previous asbestos exposure.^23^ Lack of awareness about the disease may further hinder nexus determination: because mesothelioma is rare, not all health professionals recognize it or its occupational link to asbestos. The insured’s contribution category at the time of the medical examination corresponds to their most recent contribution to social security. Thus, the INSS operating system does not allow classification of occupational etiology for insured individuals who were previously employees but, at the time of examination, were registered as self-employed, voluntary contributors, or domestic workers.

Because this study relied on secondary data, the researchers could not control data quality to ensure complete accuracy. The inherent limitations of administrative data from INSS information systems must also be considered. Finally, part of the study period overlapped with the COVID-19 pandemic, which may have restricted access to health services and thus to diagnosis as well as access to social security and, consequently, to benefits.

## CONCLUSIONS

This study analyzed social security disability benefits granted for mesothelioma in Brazil between 2019 and 2021. We found a nearly equal number of benefits granted for pleural and peritoneal mesothelioma, in contrast to the literature, which reports a clear predominance of the pleural form. This pattern may reflect longer survival among women with peritoneal mesothelioma — allowing multiple benefits to be granted to the same individual — along with possible diagnostic error. In addition, only 1 benefit was classified as an occupational disease, suggesting substantial underreporting and highlighting gaps in the documentation of occupational history and in clinical awareness of the disease.

Training health professionals to recognize mesothelioma and to establish the work-related causal nexus is essential to safeguard affected workers’ labor and social security rights. This need is especially urgent because mesothelioma incidence in Brazil is expected to rise in the current and coming decades due to the country’s historical extraction and use of asbestos and the disease’s long latency.
